# Using Fragment-Based Approaches to Discover New Antibiotics

**DOI:** 10.1177/2472555218773034

**Published:** 2018-06-20

**Authors:** Bas Lamoree, Roderick E. Hubbard

**Affiliations:** 1 YSBL, Department of Chemistry, University of York, Heslington, York, UK; 2 Vernalis Research, Granta Park, Abington, Cambridge, UK

**Keywords:** antibiotics, fragment-based lead discovery

## Abstract

Fragment-based lead discovery has emerged over the past two decades as a successful approach to generate novel lead candidates in drug discovery programs. The two main advantages over conventional high-throughput screening (HTS) are more efficient sampling of chemical space and tighter control over the physicochemical properties of the lead candidates. Antibiotics are a class of drugs with particularly strict property requirements for efficacy and safety. The development of novel antibiotics has slowed down so much that resistance has now evolved against every available antibiotic drug. Here we give an overview of fragment-based approaches in screening and lead discovery projects for new antibiotics. We discuss several successful hit-to-lead development examples. Finally, we highlight the current challenges and opportunities for fragment-based lead discovery toward new antibiotics.

## フラグメントベースのアプローチを使用した新たな抗生物質の発見

創薬プログラムにおける新規リード候補化合物を生み出すことに成功したアプローチとして、過去20年間でフラグメントベースのリード化合物発見法が浮上してきた。従来のハイスループットスクリーニングに勝る2つの主な利点は、より効率的な化学空間のサンプリングおよびリード候補化合物の物理化学的性質に対するより厳しいコントロールである。抗生物質は、効力および安全性といった性質について特に厳しい必要条件を課された薬物クラスである。新規抗生物質の開発は非常に減速しており、現在使用可能なすべての抗生物質に対して耐性が進化している。本稿では、スクリーニングにおけるフラグメントベースのアプローチおよび新たな抗生物質を得るためのリード化合物発見プロジェクトの概要を説明する。ヒットからリードへの開発の成功例をいくつか議論する。最後に、新たな抗生物質を得るためのフラグメントベースのリード化合物発見法の現在の課題と機会を強調する。

## 신규 항생제 발견에 대한 단편기반 접근법

단편기반 선도물질 발견(FBLD)은 지난 20년 사이 의약품 발견 프로그램의 신약 후보물질에 대한 성공적인 접근법으로 등장했다. FBLD는 기존의 처리량이 많은 검사 대비 화학공간의 효율적인 표본추출 및 신약 후보물질의 물리화학적 물성에 대한 엄격한 관리 등 2가지 이점이 있다. 항생제는 효능 및 안전에 있어 특히 엄격한 물성 요건을 만족하는 의약품을 일컫는다. 신항생제 개발의 속도가 너무나 더디어서 현재 상용화된 모든 항생제 약품에 내성이 생긴 상황이다. 따라서 본 연구는 검사 및 선도물질 발견 프로젝트의 신항생제에 대한 단편기반 접근법을 개략적으로 살펴보고자 한다. 본 연구는 유효물질-선도물질 개발 성공 사례를 논하였으며 마지막으로 신항생제에 대한 FBLD의 현재 도전과제 및 기회를 살펴보았다.

## 使用基于片段的方法发现新抗生素

在过去二十年中，基于片段的先导化合物的发现已经成为在药物发现项目中产生新型候选先导化合物的成功方法。其与传统高通量筛选相比的两个主要优势为更高效的化学空间取样和对候选先导化合物理化性质的更严格控制。抗生素是一类对疗效和安全性要求特别严格的药物。新型抗生素的研发速度已经放缓，以至于现在已经出现了针对每种可用抗生素药物的耐药性。在此，我们概述了新抗生素筛选和先导化合物发现项目中基于片段的筛选方法。我们讨论了几个从标靶化合物到先导化合物的成功研发示例。最后，我们强调目前在基于片段的新型抗生素先导化合物发现方面所面临的挑战和机遇。

## Introduction

Antibiotic resistance (AR) has grown into a major global health problem over the past three to four decades. Over the same period, many warnings about the dangers of emerging AR have been issued, but these have not yet resulted in improvement of the current treatment options for bacterial infections. Only in the last decade or two have national and international agencies (like the European Centre for Disease Prevention and Control [ECDC] and World Health Organization [WHO])^1,2^ proposed and implemented actual policy changes to address AR, such as strengthening prudent use of antibiotics (especially in veterinary medicine), improving surveillance and diagnostics, and increasing awareness of the need for the development of new antibiotic drugs.

The incidence of AR is increasing according to recent reports.^3,4^ Of particular concern is resistance to last-resort drugs like carbapenems, cephalosporins, and polymyxins in hospitals. There are also hints of transferability of multiple resistance genes outside the clinic.^5,6^ Lastly, the incidence of multi-drug-resistant *Mycobacterium tuberculosis* infections outside the clinic is also increasing.^7^ Tuberculosis (TB) is often discussed separately because of its differing disease and treatment characteristics.

One of the biggest current problems is that the clinical pipelines do not contain innovative new compounds to address this rising AR.^8,9^ This is often attributed to low incentives for development (any new antibiotic would be used as little as possible to avoid the generation of resistance)^10^ and technical hurdles compared with other diseases (discussed later in this review).^11^ The most recent new class of marketed broad-spectrum antibiotics (oxazolidinones) was discovered in the early 1980s,^12^ and the most recent first-in-class narrow-spectrum antibiotic to be marketed (anti-TB synthetic diarylquinoline) was discovered around the turn of the millennium.^13^

This somewhat bleak picture of the current state of antibiotics development reveals the need for increased basic research efforts and antibiotic discovery programs that can feed into the clinical pipeline. Current research does offer many promising new concepts and ideas to work with. Some examples are systematic screening for inhibitors of apparently nonessential genes as antibiotic adjuvants (β-lactamase inhibitors are a classical example) (reviewed in Wright^14^), increased understanding of mechanisms of bacterial membrane influx and efflux,^15,16^ and renewed interest in natural products using modern synthetic^17–19^ and bioinformatic tools.^20–22^

The last two decades have also seen technical advances in structure-based drug discovery,^23^ in particular a variety of developments in biophysical techniques.^24^ These advances have supported the evolution of the methods of fragment-based lead discovery (FBLD), for which there have been many reviews.^25–27^ There is a simple rationale behind the testing of fragment-sized compounds, typically with molecular weights of 120–250 Da: fragments cover a large part of theoretically possible atom configurations per molecule because they contain fewer atoms, and because of their small size, they are more likely to bind to a target. Thus, a well-designed, diverse fragment library contains about a thousand-fold fewer compounds than the average high-throughput screening (HTS) library and covers a considerably larger chemical space. However, fragments bind with a much weaker affinity (useful fragments have a dissociation constant, K_D_, of up to 2–3 mM) than usual lead compounds, and screening relies on the high sensitivity, robustness, and throughput of known and new techniques to both identify and characterize fragment binding. Once the binding of a fragment is confirmed, some initial structure–activity relationships (SARs) can be explored by the purchase of compounds that are similar to the fragment or contain substructures of the fragment (sometimes called SAR by catalog). This can increase confidence in the validity of the fragment hit, but in general, the process of growing the fragment to a lead compound is challenging without structural information generated by either crystallography or nuclear magnetic resonance (NMR) experiments. The structure of the fragment bound provides ideas about how to evolve the structure of the fragment, initially by systematic exploration of SARs, making small changes to the core scaffold. For the development of the fragment, there are three general strategies, all greatly benefiting from structural information and usually supported by biophysical methods to confirm and characterize binding: (1) fragment growing, which increases potency by optimally engaging the binding pocket, based on structural and/or SAR information; (2) fragment linking; and (3) fragment merging, in cases where there are two molecules binding close to each other.

While the main goal of hit-to-lead optimization is to increase the affinity or activity of a series, many researchers now try to optimize every single atom addition, guided by a metric called ligand efficiency (LE),^28^ which is the average binding energy of the molecule per ligand nonhydrogen atom (i.e., [–RT ln(K_D_)]/[heavy atom count]). All in all, the two core concepts of structure-guided and LE-optimized lead design leave relatively little room for serendipity in the FBLD process, but instead try to rationally optimize the chances and opportunities of finding lead compounds with novel scaffolds, favorable physicochemical properties, and target selectivity.

In this mini-review, we first summarize the key features of the techniques that are used in fragment-based discovery. The techniques are primarily considered for their use in screening to identify fragments, but the same methods can be used throughout the optimization process. We then summarize some of the examples published where the methods have been used in antibiotic discovery projects.

## Screening Methods in FBLD

A wide range of biophysical, structural, and biochemical assays have been used to identify fragments that bind to a protein target. Although some targets can have highly enclosed binding sites where fragment-sized molecules bind with a K_D_ below 1 µM (such as G-protein-coupled receptors), most fragment screening campaigns need to identify compounds that bind with affinities in the hundreds of micromolar to low micromolar range. This places particular constraints on the screening approach—requiring high solubility for the ligands, high sensitivity of the detection method, and for the assay not to be liable to interference from the high concentrations of fragment that are used. Most screening campaigns use biophysical methods, usually with an orthogonal method used for the validation and characterization of binding. There have been extensive reviews of the different fragment screening methods.^26,29,30^ The following is a brief summary of the main characteristics and considerations for each of the screening approaches. Key points for consideration of each method are also represented in **Table 1**.

**Table 1.** Summary of Main Features of Most Widely Used Fragment Screening Methods.

Technique	Throughput^a^	Protein^b^ Consumption	Lower Affinity Limit (mM)^c^	Main Limitation	Notes
Ligand-observed NMR	High	High	10	No direct structural information	
Protein-observed NMR	Low	High	5	Usually limited to proteins <35 kDa	
SPR	Moderate	Low	0.5	Requires protein immobilization	Low false-negative rate
TSA	High	Low	0.1	Insensitive	High false-negative rate
Biochemical assay	High	Low	0.1	Many ways of interference	Direct functional information
Crystallography	Moderate	Moderate	No limit	Requires high-quality crystals	Low false-positive rate
WAC	Low	Low	1	Requires protein immobilization	Low false-negative rate
MST	Moderate	Low	0.5	Requires protein labeling	

The comments are somewhat subjective and reflect the experience of the authors but summarize the comments made in the text. All techniques depend on the expertise of the user, particularly in recognizing artifacts leading to false-positive or false-negative results. In addition, the limitations are affected not only by the sensitivity of the detection method but also by compound behavior (solubility and aggregation).

a Throughput depends on the system and the instrumentation available, but “high,” “moderate,” and “low” are for many hundreds, tens, or a few compounds per day.

b For protein consumption, “high,” “moderate,” and “low” are where a screen of 1000 fragments would require many tens, single-digit, and below 1 mg of protein in most cases.

c The affinity limit, presuming that the compounds have unlimited solubility, is approximately the lowest detection limit for the technique. Note that ITC is not used for fragment screening—the protein consumption is too high, the experiment takes too long, and the binding of some fragments is entropically driven, so they would be missed.

### Nuclear Magnetic Resonance

There are a wide variety of NMR experiments where the spectra obtained are sensitive to the binding of a ligand to a protein. There are two main classes of experiments—protein observed, which detect changes in the spectrum of the protein, and ligand observed, which detect changes in the spectrum of the ligand, both of which are briefly introduced below and described elsewhere in more detail.^31–33^

Ligand-observed spectra for fragment screening are usually acquired with a large molar excess of ligand over the protein—typically with the protein at 10 µM and the ligand at 500 µM. The three most widely used experiments are saturation transfer difference (STD),34 water ligand observed via gradient spectroscopy (water-LOGSY),35 and Carr–Purcell–Meiboom–Gill (CPMG) experiments,36 in each of which binding is detected through a change in the spectrum of the ligand. For STD, a series of pulses are applied at the chemical shift of a core hydrophobic nucleus in the protein—this energy is transferred through the protein, to the ligand, and persists when the ligand dissociates into solution. This results in a difference in the spectra measured for the ligand with and without protein saturation. In water-LOGSY, the energy is transmitted between water molecules and ligand molecules. The efficiency of the transfer depends on the tumbling speed of the ligand molecules, giving differential signals for free and bound ligands. The CPMG experiment more directly measures the tumbling time of the ligand, which will be different when bound or free. These ligand-observed experiments give no indication of the site of binding, and because of the high concentrations used, it can be possible to obtain false-positive results from very weak nonspecific (and often superstoichiometric) binding. For this reason, a competitive step is usually included to check for changes to the fragment binding signal when a known ligand binds to the site of interest. In addition, the ligand-observed methods require exchange rates (approaching the diffusion limit) that allow the excess ligand in a sample to bind at least once to the protein within the timescale of the experiment, and so can miss high-affinity compounds. Also, the different physical bases of the NMR experiments can give rise to artifacts, and for this reason, it can be prudent to require positive signs of competitive binding in all three NMR experiments.37 The advantages of these ligand-observed methods are that the protein does not need isotopic labeling, there is no limit on the size of the protein, and the spectra that are obtained confirm that both the protein and the fragment are intact and in solution. The main disadvantage is the large amount (typically tens of milligrams) of protein required.

The most widely used protein-observed NMR experiment is heteronuclear single-quantum coherence (HSQC), which was used in the first published fragment-based discovery project from Abbott.^38^ Transfer of signal between ^1^H and ^15^N or ^13^C in the isotopically labeled protein results in a spectrum where each amide or methyl group gives rise to a single peak, where the position of each peak depends on the local chemical environment, which can be affected by ligand binding. The main limitations are the size of protein that can be studied (typically 35 kDa), the need for isotopic labeling (which is difficult for proteins produced through nonbacterial expression), and the need for higher protein solubility to give sufficient signal (typically 20–100 µM). This requirement also increases the amount of protein required for screening. However, the dynamic range of HSQC measurements is quite broad, mainly limited by compound solubility, although there can be issues at low micromolar affinity, where the exchange rates between free and bound populations lead to peak broadening. HSQC can also give additional information: the pattern of peaks that shift can confirm that the fragments are binding to the same binding site and, if the spectrum of the protein is assigned, then where this binding site is. Also, as long as the ligand is soluble, it is possible to titrate and obtain a K_D_ from the size of chemical shift on the protein.

A more advanced use of NMR is to determine the binding mode of the ligand bound to the protein. This requires more extensive NMR experiments that give an assignment of which peak corresponds to which nucleus, and then collection of sets of distances between the atoms from nuclear Overhauser effect (NOE) experiments. A full collection of NOEs can give a complete structure for the protein with ligand bound. A variant is to collect limited NOE data (using an isotope-filtered NOE experiment) to identify particular ligand–protein NOEs that can be used to generate an NMR-guided model of how the ligand binds.^39^ Such protein-observed NMR experiments underpinned the fragment discovery work at Abbott.^38,40,41^

### Surface Plasmon Resonance

Surface plasmon resonance (SPR) is a method to measure the change in molecular mass when a ligand binds to a protein. The most widely used equipment uses a surface to which either the ligand or the protein is attached—the other component is then flowed over the surface. The refractive index of light shone onto the surface is sensitive to the molecular mass of what is attached. If a protein is immobilized, the increase in mass as the ligand is flowed over gives information about the association rate; if the ligand is replaced with just buffer, then the dissociation rate can be measured. The ratio of the dissociation and association rates is the equilibrium constant. The current generation of instruments is sensitive enough to detect the binding of low-molecular-mass compounds, such as fragments. The main issues are in finding the conditions and strategy to immobilize the protein to provide a homogenous surface where the protein remains folded and having the reagents (and experience) to design suitable control experiments to validate the system, as summarized in the excellent review from Giannetti.^42^

### Thermal Shift Analysis

The principle behind thermal shift analysis (TSA) is that the temperature at which a protein unfolds will be changed by binding of a ligand. In practice, a protein solution (± the ligand) is heated in the presence of a fluorescent dye—the dye binds to the hydrophobic surface as the protein unfolds. The advantage of the technique is that it uses relatively cheap equipment (a qPCR machine is sufficient), uses small amounts of protein, and is quite rapid. The method works well to identify ligands that stabilize a protein for crystallization^43^ and for screening suitable buffers to stabilize the protein,^44^ and has been used for screening libraries of larger compounds.^45^ TSA has been used for fragments, but there are issues with many false negatives;^46^ a weakly binding fragment does not necessarily stabilize the protein to a detectable level. However, it is a fast and economical way of screening, attractive to academic groups.

### Biochemical Assay

A biochemical assay (such as a functional enzyme assay or a binding assay, such as displacement of a fluorescently labeled probe) can be used to screen for fragments. The main requirement is that the assay is not compromised by the high concentration of ligand (and sometimes associated solvent) of ligand being used. The advantage of a functional assay over biophysical techniques is that it only reports binders that modulate function (functional relevance of hits is often unclear, especially from crystallography^47^) and can distinguish between different types of modulators if the assay is balanced properly.^48^ The review in^49^ includes comparison of biochemical and biophysical assays.

### Crystallography

There was a strong focus on high-throughput x-ray crystallography for the screening of fragments from some of the early adopters of the approach,^50^ and there have recently been significant improvements in streamlining data collection and structure determination.^51^ There is an immediate advantage in providing a model for the binding of the fragment, and the high concentrations required for soaking experiments means quite weak binding fragments can be identified. However, it does require the protein to crystallize with an accessible active site and a crystal packing resistant to moderate solvent and ligand concentrations. It often takes a number of attempts to obtain a crystal structure of a fragment binding to a protein even when it has been confirmed to bind by other techniques.^29^

### Isothermal Titration Calorimetry

An isothermal titration calorimetry (ITC) experiment measures the heat (enthalpy or ΔH) that is released or taken up when a ligand binds to a protein, and the titration gives the equilibrium binding constant from which the entropy component (ΔS) can also be determined. As long as there is some heat change on binding, the technique is extremely robust and can be used to detect and characterize binding for proteins that are difficult to assay in other ways. Importantly, it can confirm the stoichiometry of binding. However, it is quite expensive in protein and so is not used for screening (see the survey on screening methods^25^). It also has high requirements for the solubility of the ligand (if titrated).

### Other Ideas and Approaches

Many other approaches and technologies have been suggested and used for detecting low-affinity interactions. In general, no particular technique can be regarded as a “best-in-class” solution, but it is useful to be aware of and consider all available options. Some of these other methods are proprietary, such as the capillary electrophoresis method used by Selcia,^52^ the target-immobilized NMR screening (TINS) method of ZoBio,^53^ and weak affinity chromatography (WAC).^54^ Mass spectrometry is limited to situations where the ligand binds with affinities better than approximately 100 µM, but has been applied successfully with covalently bound fragments,^55^ with the “tethered” fragments approach of Sunesis,^56^ and more generally by NovAliX.^57^ Another recently developed biophysical technique that has been successfully applied by some to FBLD is microscale thermophoresis (MST) (Nanotemper GmbH^58^). In this approach, the mobility of molecules (observed from a fluorescent label) along thermal gradients (thermophoretic mobility) is used to identify changes in molecular hydration and thus molecular interactions.

## General Comments on Fragment Screening

There is much debate about the “best” technique to use for fragment screening (e.g., see the Practical Fragments blog^25^). All the techniques have their pros and cons (see **Table 1**)—protein-observed NMR requires labeling and has a size limit, ligand-observed NMR requires large amounts of protein, x-ray crystallography requires a suitable crystal system, and SPR (and MST) requires effective labeling that does not affect function. Each of the techniques also has particular requirements in terms of the solubility of the protein and the solubility of the ligands screened and can robustly detect different dynamic ranges of binding affinity. There are occasionally studies that discuss the different hits obtained from fragment screening with different techniques (e.g., Schiebel et al.^59^). However, if the limitations of each technique are taken into account (sensitivity, buffers, solubility, etc.), then the same hits should be obtained.^49,54^ One should take caution if only taking the intersection of hits—this will identify the most robust binder but means the diversity of hits is vulnerable to the least reliable method. As mentioned before, hit validation by orthogonal techniques will filter out some false positives when those emerge from limitations of the techniques (e.g., compound intrinsic fluorescence in some types of biochemical assay or direct saturation in STD NMR). There are still many possible ways in which false positives survive orthogonal validation, as drug discovery practitioners became aware of relatively recently,^60,61^ but there seem to be no general rules applicable to all projects.^62,63^ In the end, a balance must be found between false positives and false negatives, depending on the goals of the project (taking into account scaffold diversity, chemical tractability, etc.).

## Examples of Fragment-Based Discovery of Antibiotics

### Biotin Carboxylase

**Figure 1.**
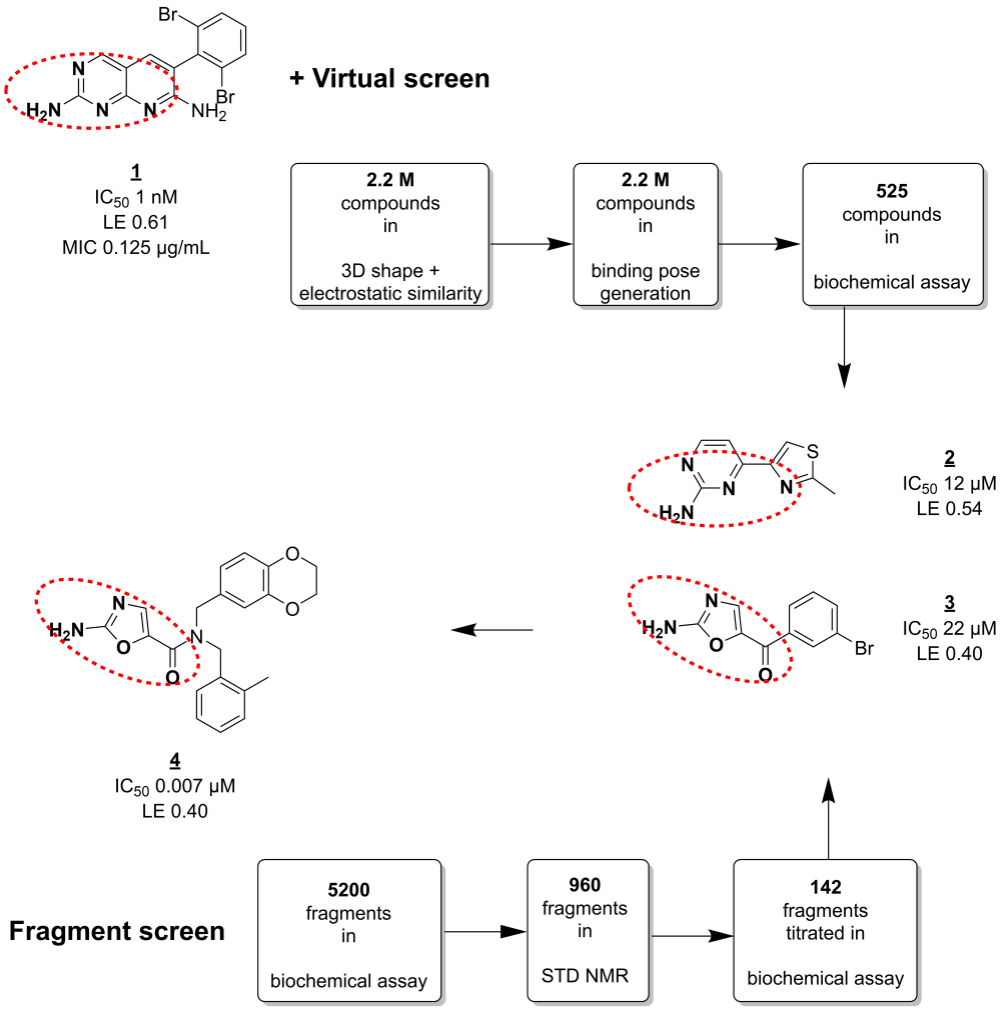
Screening against BC. Fragment and virtual screens conducted in parallel resulted in several highly ligand-efficient hits (**2**, **3**) with similar pharmacophore features to HTS hit **1**, and novel scaffolds. A number of lead series (such as **4**) were generated that showed antimicrobial activity. Red dashed circles indicate similar pharmacophore features.

Bacterial proteins without human homologs have often been prioritized as antibacterial targets. However, unbiased whole-cell screening has yielded good targets, even when homologous to human targets, as in the case of the *Escherichia coli* enzyme biotin carboxylase (BC).^64^ BC is a subunit of the bacterial acetyl-CoA carboxylase that uses adenosine triphosphate (ATP) to catalyze the first step of the reaction. The ATP binding site of BC has similarities to human kinases. Mochalkin et al. followed up on this target by using FBLD to discover potent leads with new scaffolds.^65^ They employed a cascade of three screening experiments, summarized in **Figure 1**. First, a biochemical assay that was available from their earlier work^64^ was used to screen ~5000 fragments in mixes of 10. The use of two different, moderate screening concentrations probably helped them to recognize artifacts. About 20% of the fragment mixes showed inhibition of more than 25%. Then, to identify the active component(s) of each mix, their binding to BC was measured by STD NMR. This resulted in a set of 142 fragments (3% of the library). Finally, concentration-dependent inhibition was determined by titration of the single compounds in the original enzyme assay.

The fragment screen results were complemented by virtual screening. Out of a set of 2.2 million small organic in-house compounds (including fragments), a subset was selected for 3D similarity to previously identified HTS hit **1**.^64^ Visual inspection of subsequently generated binding poses at the BC ATP binding site yielded 525 hits. This hit set was tested in the enzyme assay and contained 48 compounds (9%) inhibiting BC at 50% inhibitory concentration (IC_50_) values less than 10 µM. Furthermore, it also contained several active fragments with novel scaffolds (**Fig. 1**, such as fragment **3**).

Several of the most potent hits (such as **2**) had pharmacophore features in common with the earlier identified HTS hit **1**, and the common binding mode was confirmed from co-crystal structures. This information was used to generate a number of lead series. For example, fragment **3**, a hit from both the fragment and virtual screen, could be optimized into a potent lead **4** with bactericidal activity. As with **1**, its specific inhibition of BC could be inferred from reduced activity against a spontaneous single-amino-acid substitution mutant of BC. Interestingly, the HTS- and fragment-derived leads showed differences in properties like efflux pump susceptibility, cell penetration, and activity against different Gram-negative pathogens. In addition, the lead series were at least 70-fold selective for *E. coli* BC over a panel of more than 30 human kinases.

### DNA Gyrase

Bacterial DNA gyrase, a type II topoisomerase, is a tetrameric complex that acts on topological isomers of DNA during replication and transcription to relieve positive supercoiling of double-stranded DNA. This is an essential process and proceeds via similar mechanisms in all domains of life. There are several classes of successful bacterial DNA gyrase inhibitors (e.g., quinolones, aminocoumarins, and novel bacterial type II topoisomerase inhibitors [NBTIs]) both on the market and under development, targeting either the ATPase domain (GyrB) or the DNA cleavage domain (GyrA).

**Figure 2.**
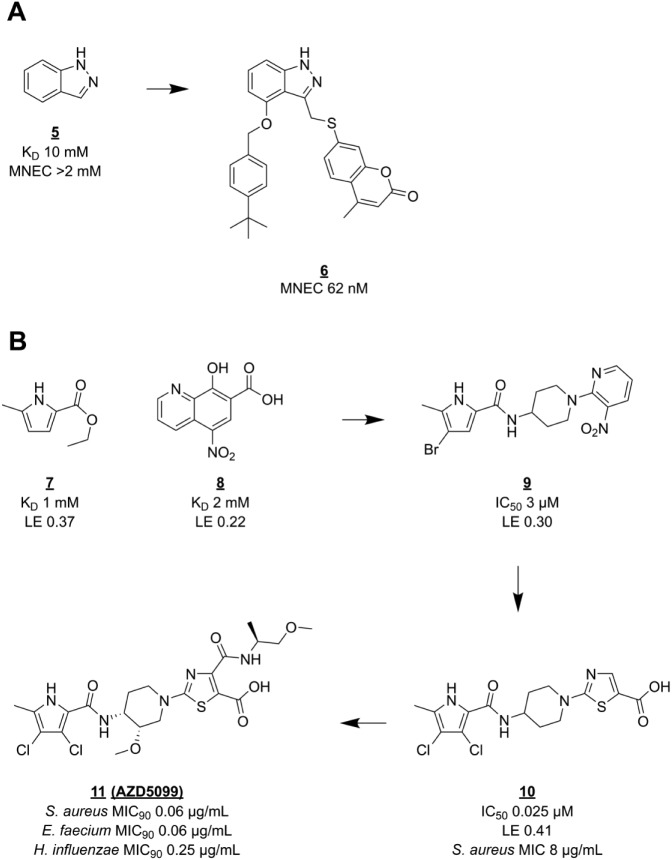
Hit-to-lead optimization of GyrB inhibitors. (**A**) Indazole (**5**), a validated hit from a virtual screen, was optimized to lead compound **6**. (**B**) Development of pyrrole hit **7** into clinical candidate **11**. MNEC = minimum noneffective concentration; MIC_90_ = MIC value at which 90% of tested strains were inhibited.

There have been several fragment-based drug discovery campaigns against DNA gyrase. One of the earliest examples of FBLD^66^ used a screening cascade similar to that described for BC. First, a selection of small fragments (then called “needles”) was made from a database of available compounds by pharmacophore fitting and docking into the GyrB ATP binding site. Then, this set of 3000 fragments was tested for biochemical activity at high (0.5 mM) concentration. Finally, a set of 150 hits divided over 14 compound classes was taken to the validation stage, which included a second biochemical assay, various biophysical binding experiments (including SPR and NMR), and SAR elaboration. Further structure-guided optimization yielded lead compounds (e.g., **6**) with up to ~30,000-fold improved inhibitory activity in vitro (**Fig. 2A**).

More recently, researchers at AstraZeneca reported the discovery^67^ of pyrrolamide GyrB inhibitors and their development^68^ into clinical phase. A low-affinity pyrrole carboxylate (**7**, **Fig. 2B**) binding in the adenine pocket was identified in a protein-observed NMR screen. This fragment engages the conserved aspartate + water motif that is also exploited by **5** and other GyrB inhibitors. Fragment **7** was part of a collection of fragments of known GyrB inhibitors that was added to the generic diverse screening library. Fragment **7** was chosen as a starting point for elaboration based on its favorable LE and growth vectors. Fragment hit **8**, even though very weak and ligand inefficient, was interesting because it bound to the aminocoumarin binding region of GyrB, presenting a way for **7** to gain affinity. Based on this reasoning, a pyrrolamide library was prepared and screened in an *E. coli* DNA gyrase ATPase assay. A 1-arylpiperidin-4-yl extension (**9**) proved to give the best increase in activity but was not yet potent enough for cellular activity. Guided by crystal structures of *Staphylococcus aureus* GyrB with bound pyrrolamides, the aromatic group was replaced by thiazole 4-carboxylate for optimal interaction with the two arginine side chains close to the aminocoumarin binding region. Together with a 100-fold increase in affinity, lead compound **10** showed not only antibacterial but also bactericidal activity against *S. aureus*, as well as an *E. coli* strain with impaired drug efflux. Further lead optimization eventually focused on the stereochemistry of the piperidine ring, which influences protein–ligand interactions via the orientation of the pyrrole and thiazole rings. The clinical candidate **11** (AZD5099) was chosen for an optimal combination of pharmacokinetic and physical properties, in part conferred by an intramolecular hydrogen bond between the deprotonated carboxylic acid and the secondary amide.^68^ Unfortunately, development of the pyrrolamide lead series was stopped after the first phase I study with AZD5099.^69^

Currently, two new DNA gyrase inhibitors, both discovered during whole-cell screening, are being evaluated by Entasis Therapeutics and GlaxoSmithKline in late-stage clinical trials for *Neisseria gonorrhoeae* infections.^8^

### Cell Division Protein FtsZ

FtsZ plays an essential role in the separation of newly forming cells during bacterial replication.^70^ It is homologous in structure to eukaryotic β-tubulin, a cancer target in humans. Like tubulin, FtsZ binds guanosine triphosphate to polymerize into strands, but FtsZ is only needed for cell division and not for chromosome separation. Inhibition of FtsZ does not directly stop growth but leads to formation of long filaments or large “balloons” that eventually lyse. Development of antibacterials targeting FtsZ has been ongoing for well over a decade. Most of the reported compounds act by preventing FtsZ polymerization, destabilizing polymers, or stabilizing polymers.^71^

**Figure 3.**
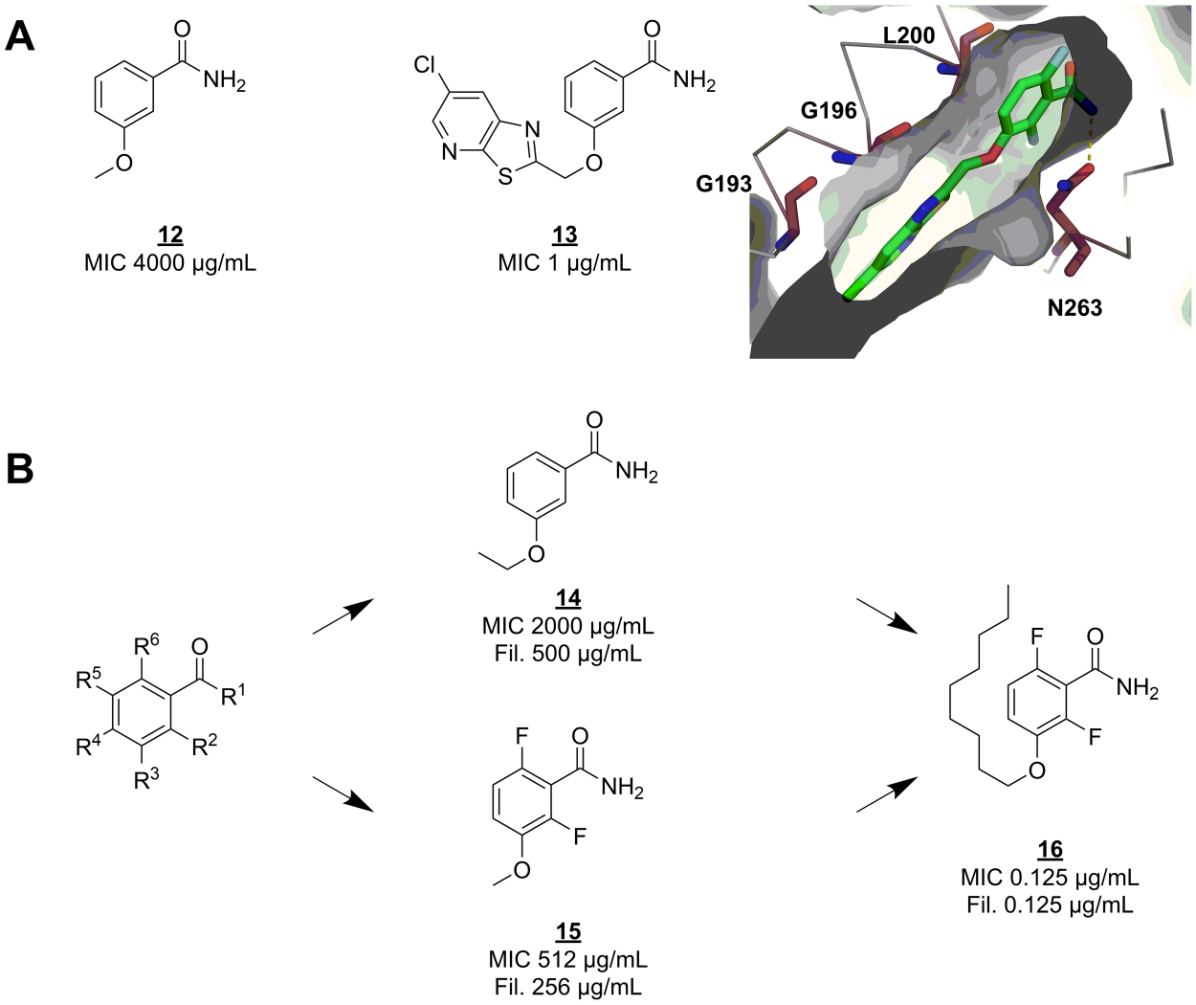
SAR exploration of the benzamide scaffold. (**A**) FtsZ inhibitor 3-methoxybenzamide (**12**) was the starting point for optimization into lead compound PC190723 (**13**), which was shown to bind in a hydrophobic cleft (PDB code 4DXD). Residues that confer resistance to **13** upon mutation are shown as raspberry-colored sticks. Hydrogen bonds are shown as dashed yellow lines. (**B**) Several substituents were placed on each R group, including combinations of two groups on different positions. Fil. = minimum concentration at which filamentation was observed.

Shortly after the discovery of the function of FtsZ,^72^ the protein was reported as the primary target for the fragment-like microbiological tool compound 3-methoxybenzamide (**12**) (**Fig. 3A**).^73^ Seeing this as a starting point for fragment-based program, Czaplewski and coworkers optimized the methoxybenzamide scaffold to a potent lead compound.^74^ PC190723 (**13**) (**Fig. 3A**) has exceptional antibacterial activity against several *S. aureus* strains, including multi-drug-resistant ones.^75^ Unlike the usual fragment screening hits, **12** was already known to be able to penetrate the bacterial cell membrane, and to cause filamentation by acting on FtsZ. In the absence of any structural information on how **12** binds to FtsZ, optimization of the benzamide scaffold was done by thorough, systematic SAR only^74^ (a strategy not favored by many FBLD practitioners today). Optimization efforts were based on minimum inhibitory concentration (MIC) and filamentation against *Bacillus subtilis*. Derivatives with variations on each possible position on the scaffold (**Fig. 3B**) were either bought or synthesized (less than five steps). This early SAR series clearly indicated that any substituents larger than a single atom on any position except R^3^ eliminated growth inhibition and filamentation in vitro. A hydrophobic group of medium size on R^3^ boosted potency up to 8000 times. Unfortunately, the nonyl group of the most potent compound (**16**) (**Fig. 3B**) is quite un-drug-like. Further optimization eventually yielded **13**, which is active against *B. subtilis* and *S. aureus* but not other Gram-positive or Gram-negative bacteria.^75^ Based on a resistance mutation profile and an apo structure of FtsZ, the researchers suggest that **12** and derived inhibitors bind to an allosteric site next to the nucleotide binding site, homologous to the Taxol binding site of tubulin. This hypothesis was later confirmed by crystallography^76^ (**Fig. 3A**), and this binding mode was shown to promote FtsZ polymerization and stabilize FtsZ polymers.^77^

This compound class is one of the most promising candidate antibiotics, even though it has a narrow spectrum (*S. aureus*, including drug-resistant strains) and has proven difficult to optimize in terms of pharmacokinetic properties.^78^ It is currently under active preclinical development by Taxis Pharmaceuticals.

### EthR

Isoniazid and ethionamide are anti-TB drugs targeting the same component of the mycolic acid synthesis pathway of mycobacteria. Because effective use of ethionamide requires high doses associated with liver toxicity, a way to lower the required dose would be an attractive therapy. Isoniazid and ethionamide have different resistance profiles because they are prodrugs activated by different bacterial enzymes. Ethionamide is activated by EthA.^79^ The *ethA* gene is regulated by the transcriptional repressor EthR.^80^ Therefore, several drug discovery projects have searched for EthR inhibitors as adjuvants for ethionamide. EthR is a relatively new anti-TB target with promising early inhibitors, as will be described. It will be interesting to see whether EthR inhibitors can be developed into therapies, as the role of EthA in ethionamide activation is not yet fully understood, as suggested by recent reports of redundant mechanisms.^81–83^

**Figure 4.**
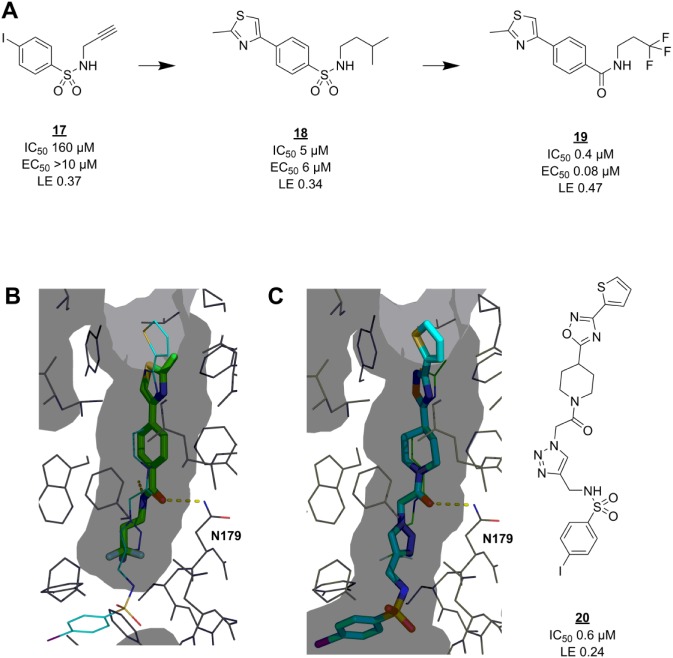
Fragmentation approach for EthR inhibitors. (**A**) Click reaction component **17** can be seen as a fragment with weak activity. It was successfully grown via virtual (**18**) and then actual medicinal chemistry to lead compound **19**. (**B**) Crystal structure (PDB code 4M3B^86^) showing the binding mode of **19** (green sticks) in the *M. tuberculosis* EthR allosteric pocket (grey surface representation). The binding pose of **20** (cyan thin lines) in the same pocket (PDB code 3O8H^85^) is overlaid. (**C**) Crystal structure (PDB code 3O8H) showing the binding mode of **20** (cyan sticks) in its binding pocket (grey surface). The binding pose of **19** (green thin lines) in the same pocket (PDB code 4M3B) is overlaid. Hydrogen bonds are shown as dashed yellow lines. EC_50_ = concentration of EthR ligand at which *M. tuberculosis* growth in macrophages is inhibited by 50% by ethionamide at 1/10 of its MIC, determined according to a standard procedure.^106^

EthR has a deep, narrow, hydrophobic pocket that facilitates allosteric deactivation upon compound binding. Researchers at the Pasteur Institute found several active hits among a small set of compounds selected by pharmacophore modeling.^84^ After attempting to expand the hits inside the binding pocket by in situ click chemistry,^85^ they took one of the click reaction components, **17** (**Fig. 4A**), as a starting point for fragment-based hit-to-lead optimization.^86^ Although extension of the original hits with this structure did not increase the potency, **17** does display weak potency on its own. The fragment was found to bind near the bottom of the binding cavity, making the critical hydrogen bond to N179 with its sulfonamide group. Based on this binding mode, a set of 61 × 16 combinations of commercially available 4-substituted benzenesulfonyl chlorides and amines (respectively) was generated and screened in silico. The resulting SAR data were validated in vitro to yield **18** with 32-fold improved inhibition of EthR DNA binding. Encouragingly, the in vitro activity of **18** was matched by its ex vivo ability to boost the antibacterial effect of ethionamide at 1/10 its MIC. The binding mode of **18** was also confirmed by crystallography to match the predicted one. Further optimizations included replacement of the sulfonamide group with an amide that picks up an additional hydrogen bond interaction with N176, leading to a lead compound (**19**) with excellent LE and higher activity than its originator, **20**. In this example, the fragmentation approach has worked well despite **17** adopting a different binding mode (**Fig. 4B**) than expected (from that of **20**, **Fig. 4C**) and also lacking the previously identified core scaffold.^84^

**Figure 5.**
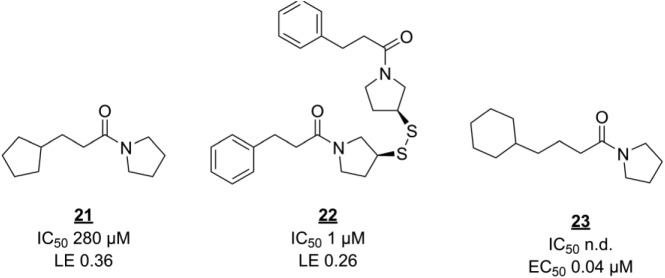
Fragment screening derived inhibitors of EthR. n.d. = not determined.

Fragment screening against EthR has produced another interesting series of compounds. Noting that the allosteric pocket of EthR is hydrophobic, Surade and colleagues used TSA to identify 86 hits from a library of 1250 fragments, some of which stabilize EthR by more than 5 °C.^87^ The hits were validated using orthogonal biophysical techniques. As mentioned earlier, it is not trivial to select the best primary and secondary screening techniques and to interpret their combined data, as each technique has its own false-positive or false-negative rates and can be more suitable for certain screening subjects or libraries than others.^88^ In this case, SPR validation data agreed well with the primary screen, with only 1 out of 45 negative controls (nonhits from TSA) showing up as an inhibitor in SPR. Only slightly more than half of the 86 TSA hits were identified as inhibitors, perhaps because of the large difference in assay concentrations (10 mM in TSA vs 0.5 mM in SPR). Several validated hits were soaked into EthR crystals to elucidate their binding modes. Interestingly, three hits contained similar arylsulfone scaffolds, as discussed in the previous example, with their sulfone groups interacting with N179. A fourth hit, **21** (**Fig. 5**), however, was more interesting because of its ability to occupy a second subpocket in the crystals, as well as the hydrophobic cavity simultaneously. Linking the two slightly modified molecules together to form **22** dramatically decreased the IC_50_. However, notably, the in vitro antibacterial potencies of these two compounds are identical, showing that straightforward optimization of binding strength and LE is not always an effective strategy. Indeed, further fragment merging efforts monitored solely by biophysical techniques were unsuccessful.^89^ The authors suggest that decreased compound membrane permeability might explain the discrepancy. Instead, SAR exploration around hit **20**, while staying within fragment size range, monitored by ITC as well as bacterial growth assays, yielded submicromolar ethionamide boosters such as **23**.^90^

### β-Lactamase

Evolution of plasmid-based β-lactamases to confer resistance to standard antibiotics was one of the first signs of a new era in healthcare. As the problem grew, inhibitors of β-lactamases (another example of adjuvants) were being used in hospital settings but not to great effect.^91^ First-generation inhibitors like the natural product clavulanic acid, themselves β-lactams, quickly lost effectiveness due to resistance and narrow activity spectrum. Another class of β-lactamase inhibitors is boronic acids. Although their effects were noted since the 1980s and gained interest from researchers, no boronic acids entered clinical use until very recently, with the Food and Drug Administration’s (FDA) approval of a combination therapy of vaborbactam with a carbapenem for urinary tract infections.

**Figure 6.**
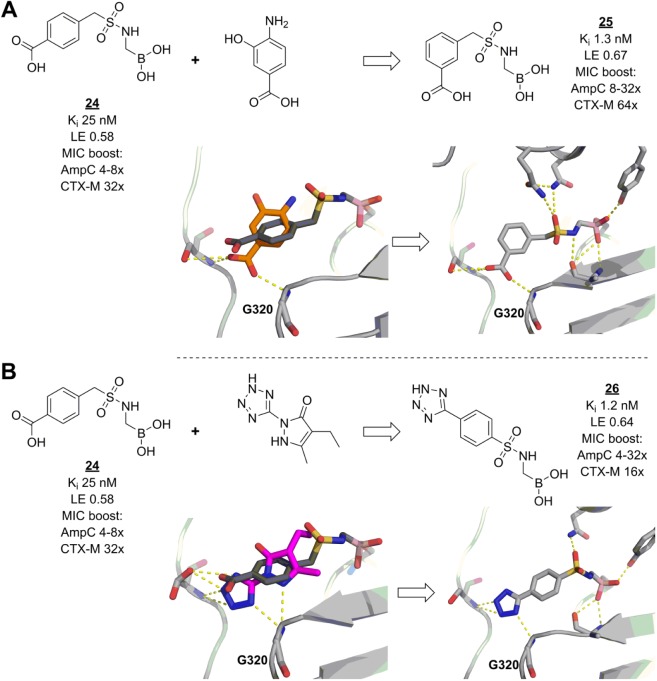
Fragments guide lead derivatization of β-lactamase inhibitors. (**A**) Lead compound **24** (dark grey) was successfully modified to **25** (light grey) to pick up an extra hydrogen bond with residue G320 of *E. coli* AmpC, inspired by a fragment (orange) binding pose (figures prepared using structures deposited with PDB codes 3O87,^94^ 2HDR,^107^ and 4E3I,^95^ for binding poses of **24**, the carboxylate fragment, and **25**, respectively). (**B**) The same interactions are made by **26** (light grey), modified to incorporate a tetrazole moiety, as suggested by another fragment (magenta) (figures prepared using structures deposited with PDB codes 3O87, 3GR2,^108^ and 4E3J,^95^ for binding poses of **24**, the tetrazole fragment, and **26**, respectively). Hydrogen bonds are shown as dashed yellow lines. AmpC = fold reduction in MIC of cefotaxime against a strain of *E. coli* overexpressing class C β-lactamase AmpC, when the compound was added at a 1:4 ratio of cefotaxime to β-lactamase inhibitor; CTX-M = fold reduction in MIC of cefotaxime against a strain of *E. coli* overexpressing class A β-lactamase CTX-M-14, when the compound was added at a 1:4 ratio of cefotaxime to β-lactamase inhibitor.

Sulfonamide boronic acids are another recently developed class of β-lactamase inhibitors. As with vaborbactam,^92^ early discovery and optimization of these molecules were guided by docking and modeling.^93,94^ Although several compounds in this series displayed high in vitro inhibitory activities, their effects on the antibacterial properties of β-lactamase-sensitive cephalosporins were only modest and did not follow the same trends. Reasoning that increasing β-lactamase affinities even further could solve the problem, the researchers made an in-depth analysis of possible enzyme–ligand interactions using results from virtual fragment screens and known inhibitor fragmentation approaches.^95^ Bound fragments can give great insight into the characteristics of the interactions of larger ligands, because fragment binding modes are unconstrained by distant binding pocket geometries. Thus, with encouraging modeling results, lead inhibitor **24** could be modified to optimize the conformation of its benzyl group and to pick up an extra hydrogen bond (compound **25**, **Fig. 6A**). Furthermore, fragment screens can efficiently offer insights into viable alternative chemical space, as shown by the model-guided replacement of the carboxylate of **24** by a tetrazole moiety, yielding **26**, which retains similar affinity and interactions as **25** (**Fig. 6B**). Interestingly, while both **25** and **26** were active as β-lactamase inhibitors in cellular assays, they showed slightly different profiles, probably due to their differing physicochemical properties. Most pronounced was the difference in ability of these compounds to also inhibit a class A β-lactamase, an effect to which no special effort was made during the optimization process. A close derivative of **26** also promoted the survival of infected mice when treated with a β-lactamase-sensitive cephalosporin, although to a lesser degree (65%) than could be reached with a β-lactamase-resistant carbapenem (90%).

## Discussion

Not enough novel antibiotics have entered the market to mitigate the threat of AR. It appears that the challenge of developing antibiotics is a particularly difficult one, and there have been several explanations for this: biased screening libraries (both synthetic and organism-derived natural products)^96^ and inadequate target selection, validation,^11,97^ or screening methods.^98^ Apart from these, the fact remains that antibiotics development is more difficult because of the extra barriers that bacteria present between a drug and its target, such as additional and different (from human) cell membranes, drug efflux, and metabolism systems. Possible solutions to these problems have been proposed, though most of them too recently to influence the antibiotics pipeline yet. For example, important aspects of drug efflux mechanisms have been elucidated,^99–101^ allowing more rational design of inhibitors and other antibiotics. Similarly, past successful and failed antibiotics give insight into their properties that are important for membrane permeation.^15,102,103^ However, all these aspects still put extra constraints on a drug’s properties. Additionally, to address the risk of rapid development of resistance, antibiotics must have exceptional efficacy and low toxicity. A hit might be modified so heavily to address all these issues that some of the original properties are lost, or worse, the hit properties conveying efficacy are incompatible with those needed for good permeability, stability, and safety. Though this might seem exacerbated in the case of fragment-based hits, FBLD could offer an advantage by having a higher chance of generating other lead scaffolds (which is why we prefer the term *fragment-based lead discovery*). The process from lead to candidate is much the same, by whichever method a lead was found.

Recently, different approaches to screening for antibacterials have been explored, including exploration of natural products from uncultured bacteria^104^ and target-based whole-cell screening.^98^ Here various FBLD approaches in antibiotic lead discovery have been discussed. One of the principles of FBLD is that it is target based. Given a target, FBLD can be used to efficiently generate leads with control over desired properties, such as lipophilicity and selectivity. For example, for GyrB, fragment screening was a good choice because it offered a way of finding new compounds for a validated mechanism of action. The fragment hit **7** is a feature of clorobiocin, a member of the aminocoumarin class of gyrase inhibitors, which are very potent but also have toxicity and solubility issues. The FBLD optimization strategy, using structural information and concepts such as LE, yielded a potent lead that even progressed to clinical candidate.

In the case of GyrB leads **6** and **10**, and in the cases of EthR lead **18** and β-lactamase leads **25**/**26**, it was fortunate that FLBD-guided increases in target affinity also resulted in whole-cell activity. Unfortunately, many other antibacterial discovery projects have shown that this cannot always be achieved. Especially for FBLD antibacterial projects, most of which in the early stages are carried out entirely in vitro with purified protein, it is important to keep an eye on the potential to gain whole-cell activity, as soon as reasonable progress after hit validation has been made. This was one of the key strategies for successful optimization of EthR fragment screening hit **21** into **23** with cellular potency.

In some cases, such as cell division protein target FtsZ, target-based whole-cell screening can even start from the fragment stage. The development of FtsZ inhibitor **13** is an unusual example of fragment-based projects because no screening was involved, no structural information was available, and the starting fragment **12** already showed specific activity in cells. Rigorous medicinal chemistry efforts were made applying principles important for every fragment-based project, especially the thorough characterization of the fragment scaffold. Small changes can have great impact on the activity of scaffolds, as evident for fragment **15** compared with **12** (as well as for EthR inhibitor **23** compared with **21**). However, the useful metric of LE cannot be used when MICs are the primary measure of potency. Indeed, it would not be easy to establish any SAR in these cases without a very specific target inhibition phenotype, because changes in MIC could also come from off-target effects.

There have recently been some exciting demonstrations of using fragments as probes for target identification in phenotypic screening. Off-target effects are expected because of the small size of the fragment, but it should have different affinities for the various targets, allowing the major targets to be identified. A recent example is where each of the fragments in the library is attached to a photoactivatable group—this covalently links the fragment to a target(s), which is then analyzed by mass spectrometry, together with methods of stable isotope labeling with amino acids in cells.^105^ This could essentially be described as whole-cell screening with direct detection of target binding and has generated a lot of interest. It will be interesting to see if this idea of functional screening of fragment libraries is successful in identifying new targets for antibiotic drug discovery.
